# Computational insights into synergistic mechanisms of PD-901 and 5-azacitidine targeting PTPN11 (SHP2) E76K mutation in juvenile myelomonocytic leukemia

**DOI:** 10.3389/fbinf.2026.1887328

**Published:** 2026-09-04

**Authors:** Abeer M. Al-Subaie, Balu Kamaraj

**Affiliations:** 1 Department of Clinical Laboratory Sciences, College of Applied Medical Sciences, Imam Abdulrahman Bin Faisal University, Dammam, Saudi Arabia; 2 Department of Dental Education, College of Dentistry, Imam Abdulrahman Bin Faisal University, Dammam, Saudi Arabia

**Keywords:** 5-azacitidine, cancer, inhibitor, juvenile myelomonocytic leukemia, PD0325901

## Abstract

**Introduction:**

Juvenile Myelomonocytic Leukemia (JMML) is an aggressive pediatric hematological cancer categorized by aberrant activation of the RAS/MAPK pathway. It is commonly caused by gain-of-function mutations, such as E76K in the PTPN11 protein. While 5-azacitidine (5-Aza) and the MEK inhibitor PD0325901 (PD-901) are not known direct inhibitors of SHP2/PTPN11, the reported activity of 5-Aza combined with MEK inhibition in PTPN11-mutated JMML offered a biological rationale for examining their potential structural interactions with SHP2.

**Methods:**

This study used a computational approach that included molecular docking, molecular dynamics (MD) simulations, and binding free-energy calculations using the MM-PBSA method to exploratively investigate interactions between 5-azacitidine (5-Aza) and the MEK inhibitor PD0325901 (PD-901) with wild-type and E76K-mutant PTPN11 proteins.

**Results:**

In the molecular docking study, 5-Aza was observed to bind within the active site, whereas PD-901 bound preferentially to the allosteric site. The dual-ligand simulations were used to investigate whether occupancy of these distant regions was associated with changes in PTPN11's structural and dynamic characteristics. MD simulations showed that the structures of all complexes remained stable throughout, and that the combined structure exhibited lower deviations, less residue flexibility, compactness, and more intermolecular hydrogen-bond interactions. Principal component analysis also revealed differences in the dominant conformational motions of the ligand-bound systems. MM-PBSA calculations provided comparative estimates of binding energetics and revealed promising interaction energies for all complexes, particularly in the E76K mutant system.

**Discussion:**

Overall, these computational findings suggest that the association of 5-Aza and PD-901 with distinct regions of PTPN11 may influence its structural and dynamic properties. These findings should therefore be considered exploratory and do not establish direct SHP2 binding or inhibition by either compound. Further biochemical, biophysical, and cellular studies are needed to validate these predicted interactions and determine their potential biological or therapeutic relevance in JMML.

## 1 Introduction

Juvenile Myelomonocytic Leukemia (JMML) is a rare but aggressive myeloproliferative disorder that mainly affects infants and children characterized by aberrant activation of the RAS/MAPK signaling pathway (Mayerhofer et al., 2021; Fiñana et al., 2022; Wehbe et al., 2021; Meynier and Rieux-Laucat, 2020). RAS pathway gene mutations are often responsible for this abnormal activation, especially PTPN11 mutations, which encode the SHP2 protein tyrosine phosphatase. PTPN11 mutations occur in about 35% of JMML patients (Fiñana et al., 2022; Pasupuleti et al., 2023; Zhao et al., 2023; He et al., 2023; Cai et al., 2020; De Vos et al., 2022). Gain-of-function mutations in PTPN11/SHP2, such as E76K, increase activity by activating downstream signaling pathways, including MAPK, leading to the proliferation of hematopoietic stem/progenitor cells (HSPC). These mutations contribute significantly to JMML aggressiveness by increasing SHP2 activity (Zhao et al., 2023; He et al., 2023; Zhao et al., 2021; Ramdas et al., 2022; Welsh et al., 2023; Kanumuri et al., 2022; Solman et al., 2022; Wu et al., 2021). The E76K mutation specifically alters SHP2 conformational state from inactive to active by disrupting SHP2 auto-inhibition (Zhang et al., 2025).

Currently, Hematopoietic stem cell transplantation (HSCT) is the only known curative treatment for JMML. However, there is a significant possibility of relapses after HSCT and side effects in patients with certain PTPN11 mutations (Wehbe et al., 2021; De Vos et al., 2022; Ra et al., 2024; Wu et al., 2024). There is a need for novel therapies that can inhibit aberrant RAS/MAPK signaling and reduce the number of leukemia cells (Meynier and Rieux-Laucat, 2020; He et al., 2023; De Vos et al., 2022; He et al., 2021). There are currently many different approaches to treating JMML. A commercial MEK inhibitor called trametinib has also shown promise in treating patients with relapsed or refractory JMML. (Stieglitz et al., 2021). Previous preclinical studies using KRAS and NF1-mutant mice treated with the allosteric MEK inhibitor PD-901 have shown reductions in spleen size and white blood cell counts, with resolution of anaemia and thrombocytopenia (Chang et al., 2012; Lyubynska et al., 2011). Additionally, the use of epigenetic drugs such as 5-azacitidine (5-Aza) is effective in patients with both newly diagnosed and relapsed JMML (Pasupuleti et al., 2023; Wu et al., 2024; Niemeyer et al., 2021; Cseh et al., 2015; Chang et al., 2024). Responses to individual agents, however, may be limited, which is why combination strategies that target complementary aspects of JMML biology should be investigated (Pasupuleti et al., 2023; Chang et al., 2024; Sinha et al., 2023; Pasupuleti et al., 2020; Liu et al., 2023; Wang et al., 2020).

Studying the molecular mechanisms underlying this condition, scientists have speculated that combination therapy could be an effective strategy to combat drug resistance (Pasupuleti et al., 2023; Chang et al., 2024). This is because 5-azacitidine and MEK inhibitors have different modes of action. As noted before, the hypomethylating agent 5-Aza primarily acts by reversing DNA hypermethylation, thereby restoring the expression of tumor suppressors and modulating the immune response (Wu et al., 2024; Day et al., 2024; Wang et al., 2023; Yuan et al., 2020; Li et al., 2020; Pangajavalli et al., 2021; Shen et al., 2020). Neither 5-Aza nor PD-901 has been shown to bind SHP2 protein in any experiments to date, nor are they recognized as direct inhibitors of SHP2/PTPN11. Therefore, in this study, SHP2 is not considered a confirmed direct pharmacological target of these substances. Instead, 5-Aza, with a proven therapeutic effect when combined with MEK inhibition in PTPN11-mutated JMML, provides a biologically and clinically significant setting for examining the potential for additional structural relationships with SHP2. Given the critical role PTPN11 E76K plays in JMML pathogenesis, we considered it sensible to investigate whether 5-Aza and PD-901 could exhibit plausible interaction modes with wild-type or E76K-mutant PTPN11.

In this study, Computational approaches, including molecular docking, molecular dynamics (MD) simulations, and MM-PBSA calculations, were used to explore potential structural interactions of 5-Aza and PD-901 with wild-type and E76K-mutant PTPN11. The specific focus was the effect of individual drugs and combinations, where 5-azacitidine (Aza) is used along with either PD0325901 (PD-901) or trametinib. The location of 5-Aza within the SHP2 catalytic-site region was considered an exploratory docking hypothesis rather than evidence of an established biological binding site, whereas PD-901 was studied in relation to a possible allosteric region. Molecular docking was performed to analyze the binding affinity between the drug molecules and the protein targets. Individual docking studies were conducted for each drug alone and in combination with other drugs, such as 5-azacitidine with PD-901 or trametinib. The findings revealed details of possible binding modes, key residues involved, and the mechanism by which active-site and allosteric ligands together affect protein-ligand interactions. To further examine the stability of these interactions, molecular dynamics (MD) and MM-PBSA analyses were performed to assess the structural stability and interactions over time. Based on molecular docking and molecular dynamics analyses, combination treatment with 5-azacitidine and the MEK inhibitor PD-901 will exhibit stronger interactions and greater stability than monotherapy with wild-type and E76K-mutant PTPN11.

Overall, this study provides computational insights into potential interactions between 5-Aza and PD-901 with both wild-type and E76K-mutant PTPN11. By combining molecular docking, MD simulations, and MM-PBSA analyses, we investigated predicted binding modes, interaction stability, energetic profiles, and mutation-associated changes in protein dynamics. The results are intended to generate structural hypotheses about the potential association of these compounds with SHP2, rather than to establish a direct mechanism of drug action. Further, experimental biochemical, biophysical, and cellular studies will be required to determine whether the expected interactions occur and whether they have any biological or therapeutic significance in PTPN11-mutated JMML.

## 2 Methodology

### 2.1 Dataset preparation

The 3-D structure of the PTPN11/SHP2 protein was downloaded from the Protein Data Bank (Berman et al., 2002) (PDB ID: 7JVM) (LaMarche et al., 2020). The monomeric form of the PTPN11/SHP2 crystal structure (PDB ID: 7JVM) was obtained from the PDB for all computational analyses. Prior to molecular docking, the co-crystallized allosteric inhibitor TNO155 was removed from the protein structure, along with water molecules and other non-essential heteroatoms. The monomeric structure was considered suitable for examining conformational dynamics and ligand-binding interactions in both the wild-type and E76K mutant proteins. The E76K mutant was generated by substituting glutamic acid (E76) with lysine (K76) using SWISS-PDB Viewer (Kaplan and Littlejohn, 2001). Energy minimization was then used to optimize the mutant structure prior to molecular docking and molecular dynamics simulations. The three inhibitors selected for this investigation are 5-azacitidine, PD0325901 (PD-901), and trametinib, which were identified using the PubChem database (Kim, 2016). For this study, the following compounds were used: 5-azacitidine as the active-site ligand and PD-901 and trametinib as allosteric inhibitors.

### 2.2 Molecular docking

Molecular docking analysis was performed to investigate the binding interactions and affinities between the wild-type and mutant PTPN11 proteins and the inhibitors. This molecular docking study was performed using AutoDock Vina (v.1.1.2) software (Trott and Olson, 2010). In this regard, initially, the structure of ligands in SDF format was converted to PDBQT format using the software Open Babel (v.3.1.1) (O'Boyle et al., 2011), and then their structure was optimized by the use of Avogadro (v.1.2.0) (Hanwell et al., 2012) employing the force field MMFF94 for 5,000 steps using the algorithm steepest descent method. In molecular docking, 5-azacitidine was docked into the active site, whereas PD-901 was docked into the allosteric site. To compare the allosteric docking performance of PD-901, the FDA-approved allosteric MEK1/2 inhibitor trametinib was used as a reference control. The dimensions of the active site grid box were 20 × 18 × 18 Å, and its centre coordinates were (26.528, 24.428, 24.428). For the allosteric site grid, the dimensions were 24 × 16 × 16 Å, and its centre coordinates were (23.511, 39.589, 7.104). The dual-ligand complexes were subjected to sequential molecular docking. To create the protein-ligand complex, 5-azacitidine was first docked into the active site. The top-ranked pose with the lowest predicted binding energy was chosen. Using the same docking parameters, PD-901 was then docked into the allosteric site using this complex, which had 5-azacitidine in its binding position. Favorable binding affinity, suitable binding orientation, and the lack of notable steric clashes between the two ligands were the criteria used to choose the resulting ternary complex. The top-ranked docking pose with the lowest predicted binding energy for each protein-ligand complex was selected for further analysis. Pose selection was based on the AutoDock Vina scoring function and visual inspection of the binding orientation within the predefined active or allosteric binding site. The docking results and their complexes were visualized using PyMOL 3.1 and Discovery Studio Visualizer v2.0 (DeLano, 2002; Visualizer, 2005) to observe essential interactions such as hydrogen bonding and hydrophobic interactions. The selected pose was subsequently used as the initial structure for molecular dynamics simulations.

### 2.3 Molecular dynamics simulations and MM-PBSA

Molecular docking was performed to obtain complexes between the protein and ligands, which were then used as initial structures in molecular dynamics (MD) simulations. For the MD simulations, the default parameters reported in previous studies (Gangadharappa et al., 2020; Datta and Doss, 2026; Loganathan and George Priya, 2025; Kamaraj and C, 2025; Kamaraj et al., 2025; Kamaraj, 2025) were adopted and are described in detail below. The complexes chosen for analysis include apo-structure proteins (PTPN11-WT and PTPN11-E76K), and ligand-protein complexes, namely, PTPN11-WT-Aza, PTPN11-E76K-Aza, PTPN11-WT-PD-901, PTPN11-E76K-PD-901, and combinations of these complexes, namely, PTPN11-WT-Aza-PD-901 and PTPN11-E76K-Aza-PD-901. The ligand topologies were generated using Antechamber (Wang et al., 2001) with the GAFF force field, and partial atomic charges were assigned using the AM1-BCC method. In contrast, protein topologies were generated using the pdb2gmx component of GROMACS-2019.4 (Abraham et al., 2014). The pdb2gmx module automatically assigned protonation states of ionizable amino acid residues during topology generation using standard protonation states at physiological pH (pH 7.0). During system preparation, hydrogen atoms were added appropriately. To reduce steric hindrance, all systems were first subjected to energy minimization in vacuum using the steepest-descent algorithm for up to 50,000 steps until the maximum force was less than 1,000 kJ mol^-1^ nm^-1^. The SPC/E water model was then used to place the minimized systems within a cube-shaped simulation box. The minimized systems were centred in a cubic PBC with a minimum distance of 1.0 nm between the protein surface and the box boundaries. To recreate physiological conditions, the systems were ionized at an ionic strength of 0.15 M, with the corresponding numbers of sodium and chloride ions added. Using the Particle Mesh Ewald (PME) method, long-range electrostatic interactions were handled using fourth-order interpolation and a Fourier grid spacing of 0.16 nm. Short-range electrostatic and van der Waals interactions were calculated using a cutoff distance of 1.0 nm. Neighbor searching was performed using the Verlet cutoff scheme. After energy minimization, a 2 fs integration timestep was used to equilibrate the systems in the NVT and NPT ensembles for 100 ps each. During NVT equilibration, the temperature was maintained at 310K using the V-rescale thermostat with a coupling constant of 0.1 ps. Subsequently, NPT equilibration was performed at 310 K and 1 bar using the Parrinello-Rahman barostat with a coupling constant of 2.0 ps and an isothermal compressibility of 4.5 × 10^−5^ bar^-1^. Covalent bonds involving hydrogen atoms were constrained using the LINCS algorithm. Production molecular dynamics simulations were run under periodic boundary conditions for 300 ns following equilibration. The GROMACS software package (Abraham et al., 2014) was then used to assess structural stability and conformational dynamics using root-mean-square deviation (RMSD), root-mean-square fluctuation (RMSF), radius of gyration (Rg), solvent-accessible surface area (SASA), hydrogen-bond analysis, and principal component analysis (PCA). The molecular mechanics Poisson-Boltzmann surface area (MM-PBSA) method was used to evaluate ligand-binding affinity. Using regularly spaced trajectory snapshots corresponding to the last 50 ns of each simulation, binding free energies were computed using the g_mmpbsa program (Kumari et al., 2014). This method made it easier to compare the systems under investigation and offered insights into the energetics and stability of protein-ligand interactions. To aid in reproducibility, the Supplementary Material contains the initial input structures and GROMACS molecular dynamics parameter (mdp) files used for energy minimization, equilibration, and production simulations.

## 3 Results

### 3.1 Docking analysis of aza and MEK inhibitors (PD-901 & trametinib) with wild-type and E76K-mutant PTPN11 proteins

**FIGURE 1:**
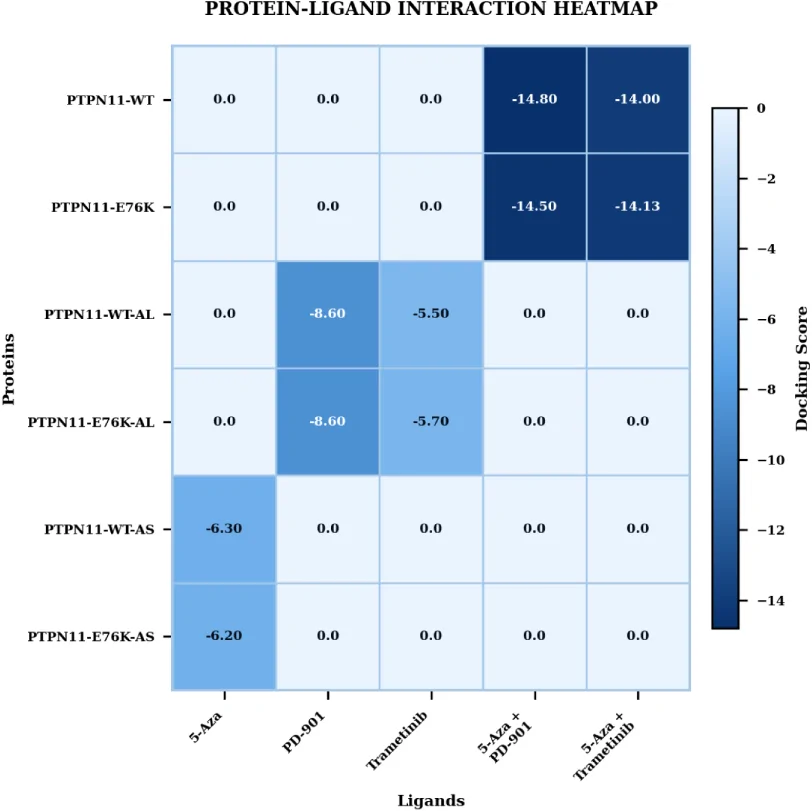
The heat map of docking scores between the wild-type and mutant PTPN11 with 5-Azacitidine and MEK inhibitors (PD-901 & Trametinib).

**FIGURE 2:**
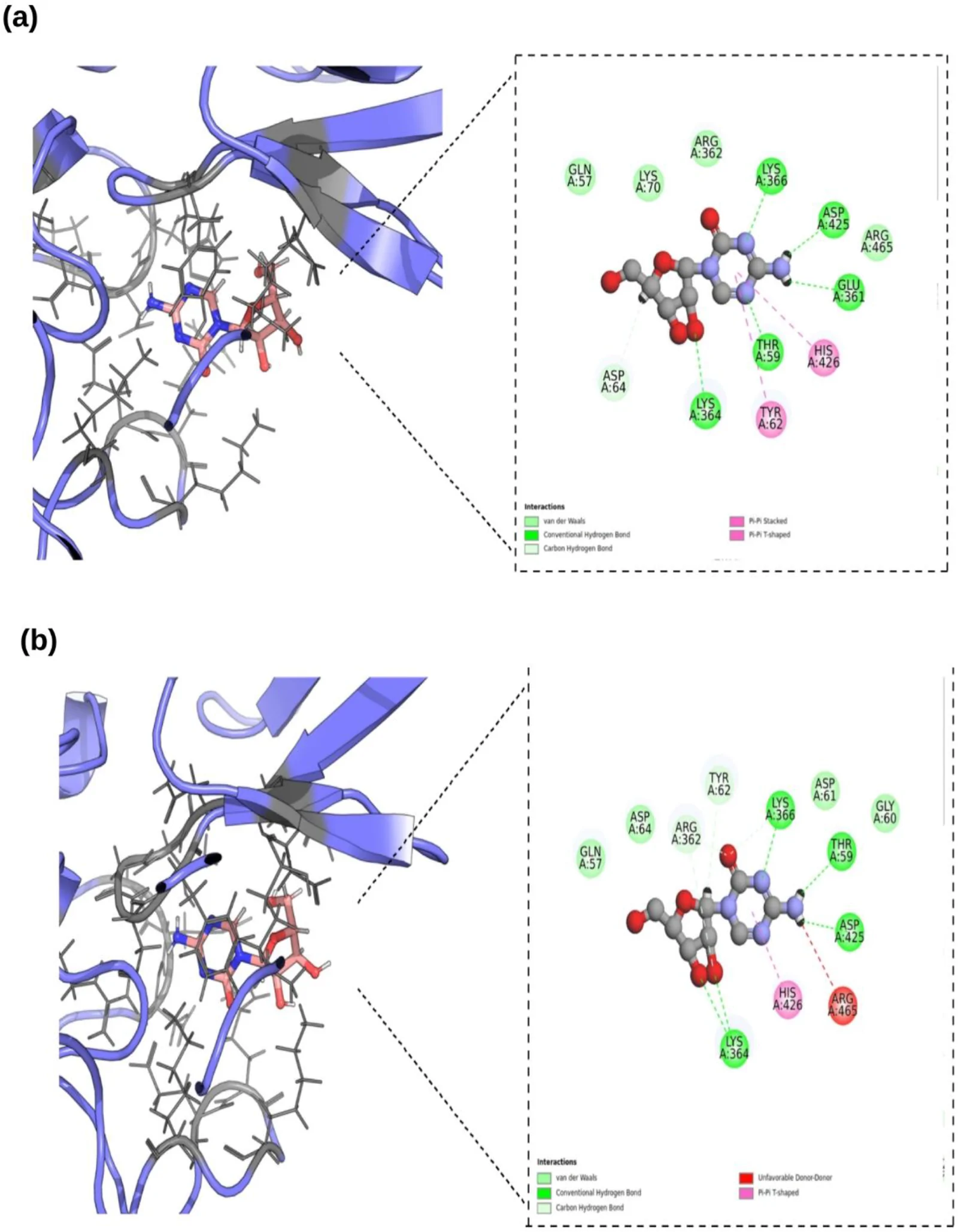
The 3-D & 2-D interaction between proteins and ligands in the active site complex: **(a)** PTPN11-WT-Aza and **(b)** PTPN11-E76K-Aza.

**FIGURE 3:**
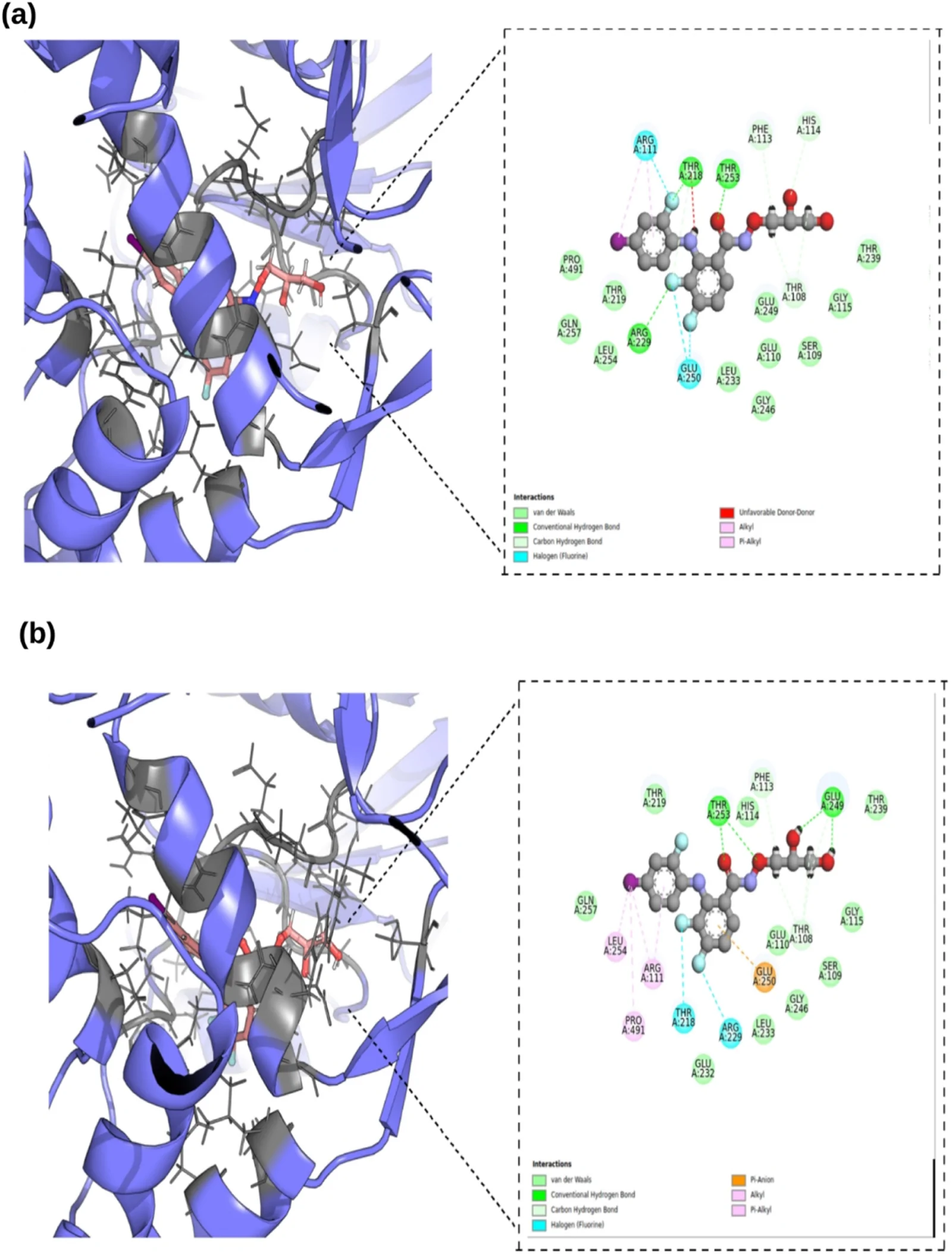
The 3-D & 2-D interaction between proteins and ligands in the allosteric site complex: **(a)** PTPN11-WT-PD-901and **(b)** PTPN11-E76K-PD-901.

**FIGURE 4:**
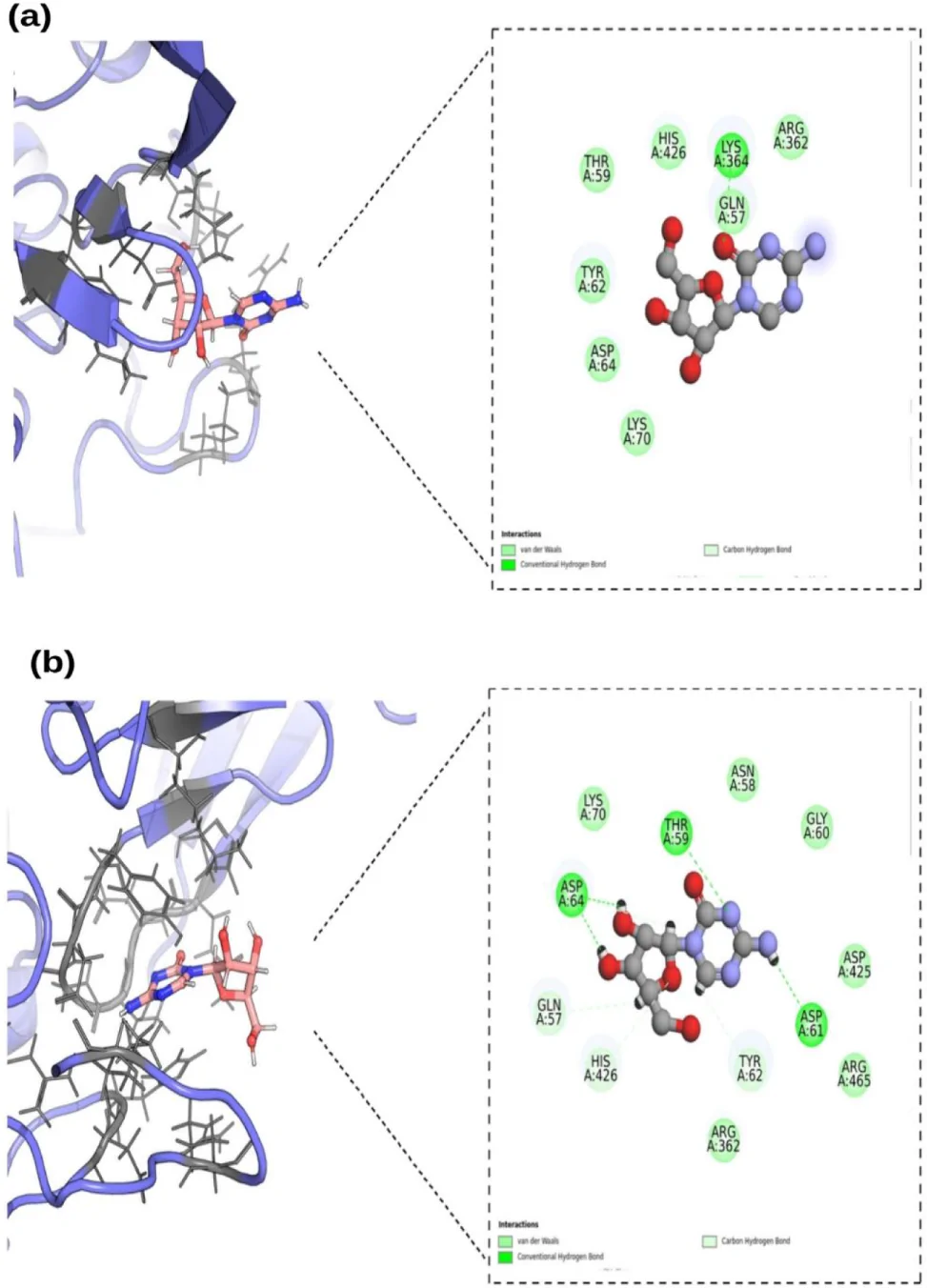
The 3-D & 2-D interaction between proteins and ligands in the active [AS] and allosteric site [AL] complex: **(a)** PTPN11-WT-Aza [AS], **(b)** PTPN11-WT-PD-901 [AL].

**FIGURE 5:**
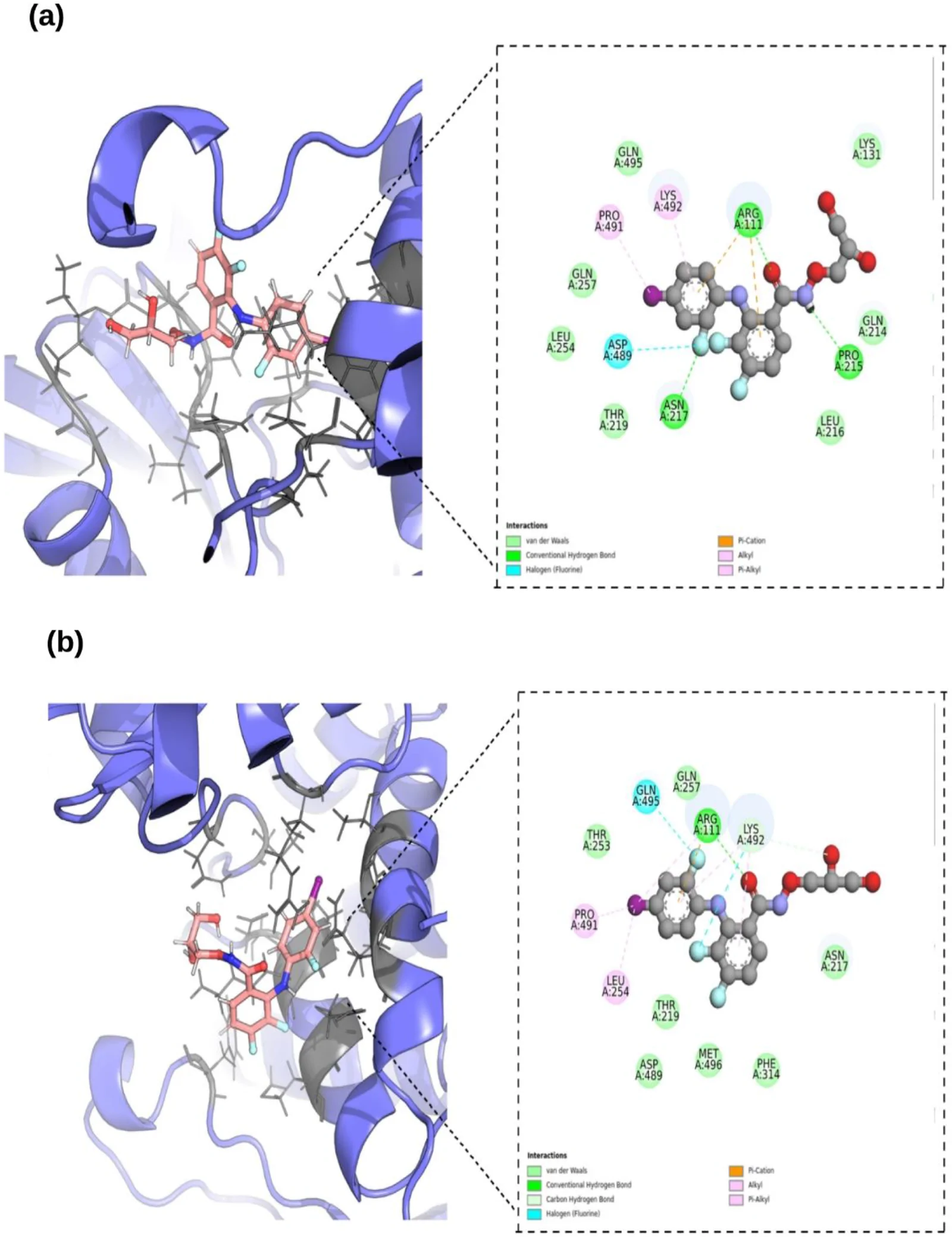
The 3-D & 2-D interaction between proteins and ligands in the active [AS] and allosteric site [AL] complex: **(a)** PTPN11-E76K-Aza [AS], and **(b)** PTPN11-E76K-PD-901 [AL].

Molecular docking analysis was performed to calculate the binding affinities and interaction profiles of 5-azacitidine (5-Aza), PD-901, and trametinib with both wild-type (WT) and E76K mutant (MT) PTPN11 proteins. Results of the molecular docking for each protein-ligand complex, including their binding energy and residue interaction sites, are shown in Table 1 and Figure 1. The PTPN11-WT-Aza complex had a docking energy of −6.3 kcal/mol at the active site (Table 1). Interacting residues in the PTPN11-WT-Aza complex are shown in Figure 2a. The PTPN11-E76K-Aza complex obtained a docking energy of −6.2 kcal/mol at the active site (Table 1). Interactive residues in the PTPN11-E76K-Aza complex are shown in Figure 2b. The PTPN11-WT-PD-901 complex obtained a docking energy of −8.6 kcal/mol at the allosteric site (Table 1). The PTPN11-E76K-PD-901 complex also showed a docking score of −8.6 kcal/mol at the allosteric site (Table 1). The interactive residues of the PTPN11-WT-PD-901 & PTPN11-E76K-PD-901 complexes were illustrated in Figures 3a,b. The PTPN11-WT-Trametinib complex had a docking score of −5.5 kcal/mol at the allosteric site (Table 1). The interacting residues of PTPN11-WT-Trametinib were illustrated in Figure 3c. PTPN11-E76K-Trametinib complex showed a docking score of −5.7 kcal/mol at the allosteric site (Table 1). PTPN11-E76K-Trametinib complex interactive residues are shown in Figure 3d. A combination of 5-Aza & PD-901 inhibitors with PTPN11-WT protein exhibited a docking score of −14.83 kcal/mol (Table 1). The combination of PTPN11-WT with 5-Aza & PD-901 complex interacting residues is shown in Figures 4a,b. The PTPN11-E76K protein, in combination with 5-Aza & PD-901, showed a docking score of −14.59 kcal/mol (Table 1). The combination of PTPN11-WT with 5-Aza & Trametinib complex interacting residues is shown in Figures 4a,b. The PTPN11-E76K with 5-Aza & Trametinib complex showed a docking score of −14.13 kcal/mol (Table 1). The combination of PTPN11-E76K with the 5-Aza & Trametinib complex interacting residues is shown in Figures 5a,b.

**TABLE 1:** Docking scores and interactive residues between the wild-type and mutant PTPN11 with 5-Azacitidine and MEK inhibitors (PD-901 & Trametinib).

Protein	Ligands	Binding site	Docking score (K/Cal)	Interactive resides
WT	5-Azacitidine	Active site	−6.3	GLN57, ASN58, THR59, ASP61, TYR62, ASP64, GLU361, ARG362, LYS364, LYS366, ASP425, HIS426, ARG465
E76K	5-Azacitidine	Active site	−6.2	GLN57, ASN58, THR59, ASP61, TYR62, ASP64, ARG362, LYS364, HIS426
WT	PD0325901	Allosteric site	−8.6	THR108, ARG111, PHE113, HIS114, GLY115, THR218, LEU233, GLU249, THR253, PRO491
E76K	PD0325901	Allosteric site	−8.6	THR108, ARG111, PHE113, HIS114, GLY115, THR218, THR219, LEU233, THR239, GLY246, GLU249, GLU250, THR253, PRO491
WT	Trametinib (reference)	Allosteric site	−5.5	ARG111, HIS114, ASN217, THR219, GLU250, THR253, LEU254, GLN257, LYS260, ASP489, PRO491, LYS492, GLN495
E76K	Trametinib (reference)	Allosteric site	−5.7	THR108, GLU110, ARG111, PHE113, HIS114, HIS116, LEU216, THR218, THR219, ARG229, LEU233, GLY246, GLU249, GLU250, THR253, LEU254, PRO491
WT	5-Azacitidine + PD0325901	Active + allosteric site	−14.83	As: GLN57, ASN58, THR59, ASP61, TYR62, ASP64, GLU361, ARG362, LYS364, LYS366, ASP425, HIS426, ARG465 AL: ARG111, PHE113, GLN214, PRO215, ASN217, THR219, GLU250, THR253, LEU254, GLN257, ASP489, PRO491, LYS492, GLN495
E76K	5-Azacitidine + PD0325901	Active + allosteric site	−14.59	As: GLN57, THR59, ASP61, TYR62, ASP64, ARG362, LYS364, LYS366, HIS426 AL: GLU110, ARG111, GLN214, PRO215, ASN217, THR219, GLU250, THR253, LEU254, GLN257, ASP489, PRO491, LYS492, GLN495
WT	5-Azacitidine + trametinib	Active + allosteric site	−14.0	As: GLN57, ASN58, THR59, ASP61, TYR62, ASP64, GLU361, ARG362, LYS364, LYS366, ASP425, HIS426, ARG465 AL: THR108, GLU110, ARG111, PHE113, HIS114, HIS116, LEU117, THR218, THR219, LEU233, GLU249, GLU250, THR253, LEU254, PRO491
E76K	5-Azacitidine + trametinib	Active + allosteric site	−14.13	As: GLN57, THR59, ASP61, TYR62, ASP64, ARG362, LYS364, LYS366, HIS426 AL: THR108, GLU110, ARG111, PHE113, HIS114, HIS116, LEU117, THR218, THR219, LEU233, GLU249, GLU250, THR253, LEU254, PRO491

WT, Wild Type; E76K - Mutant Type.

### 3.2 Molecular dynamics simulations

MD simulation approach was used to examine the structural dynamics of proteins and their interactions with ligands. The application of this method has brought about a paradigm shift in computer-assisted drug design and discovery by enabling analysis of molecular systems at the atomistic level. The current study used an MD simulation approach to observe the dynamic alterations following ligand-protein binding. Several parameters, such as RMSD, RMSF, Rg, SASA, and inter-hydrogen bonding, were calculated for both the protein and protein-ligand complex. We have used PTPN11-APO-WT, PTPN11-APO-E76K, PTPN11-WT-Aza, PTPN11-E76K-Aza, PTPN11-WT-PD-901, PTPN11-E76K-PD-901, PTPN11-WT-Aza-PD-901 and PTPN11-E76K-Aza-PD-901 complex systems for MD simulations.

#### 3.2.1 RMSD

**FIGURE 6:**
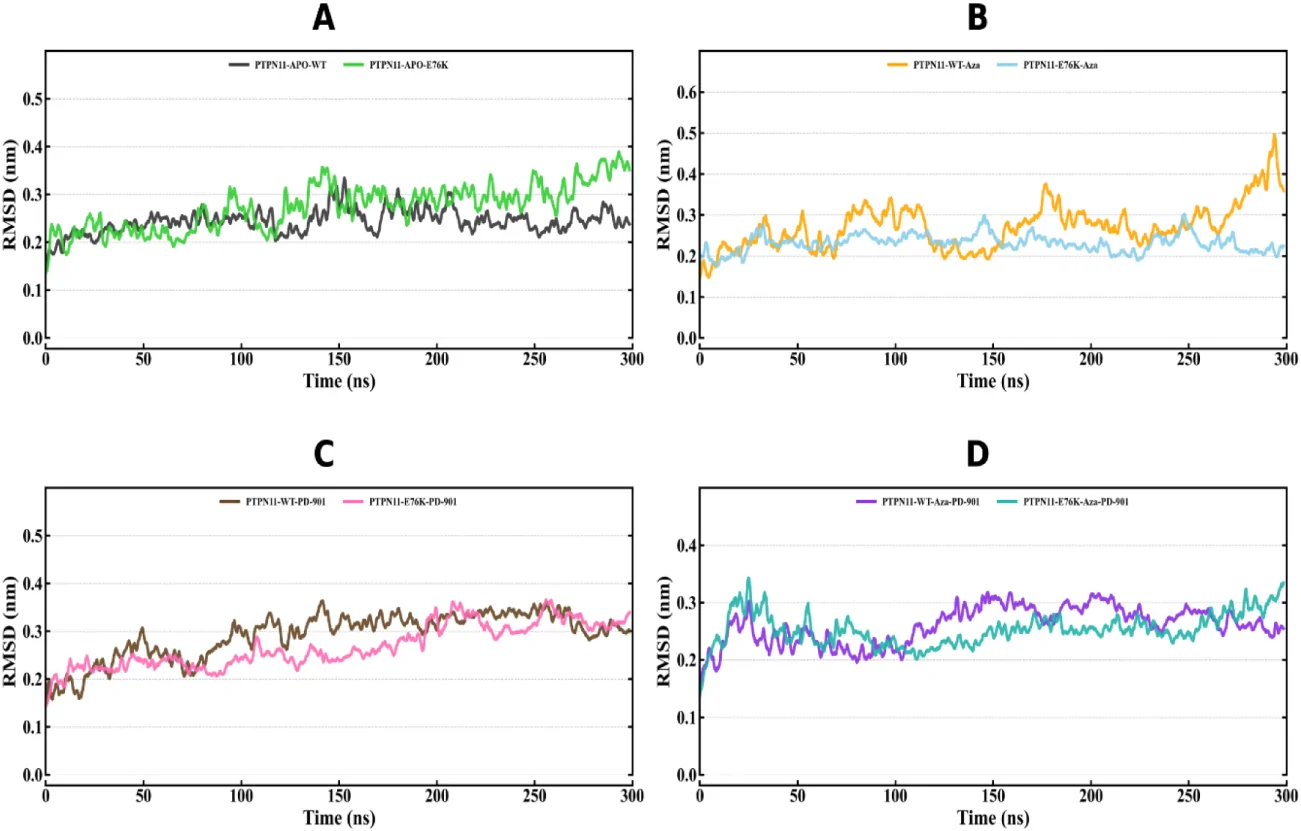
RMSD Conformational dynamics analysis over a 300 ns simulation period. **(A)** PTPN11-APO-WT Vs. PTPN11-APO-E76K; **(B)** PTPN11-WT-Aza Vs. PTPN11-E76K-Aza; **(C)** PTPN11-WT-PD-901 Vs. PTPN11-E76K-PD-901; **(D)** PTPN11-WT-Aza-PD-901 Vs. PTPN11-E76K-Aza-PD-901.

To examine the stability of the protein-ligand complexes and gain insight into the system’s behavior, RMSD values were analyzed over 300 ns (Figures 6A–D). In the RMSD plot, the PTPN11-APO-WT and PTPN11-APO-E76K structures showed similar deviations up to approximately 40 ns (Figure 6A). After that, both systems reached equilibrium within ∼50 ns and remained stable throughout the simulation time (300ns). The average RMSD values for PTPN11-APO-WT and PTPN11-APO-E76K were determined to be 0.24 ± 0.03 nm and 0.27 ± 0.05 nm (Table 2). The PTPN11-WT-Aza and PTPN11-E76K-Aza complexes showed parallel deviations up to approximately 45 ns (Figure 6B). Following this, both systems attained equilibrium and maintained their stable conformation throughout the simulation (300 ns). The average RMSD value was 0.26 ± 0.05 nm for PTPN11-WT-Aza and 0.23 ± 0.02 nm for PTPN11-E76K-Aza (Table 2).

**TABLE 2:** Average values of RMSD, RMSF, Rg, SASA, inter-hydrogen bonds, and covariance value between apo & complex wild-type and mutant PTPN11 with 5-Azacitidine and PD-901.

Complex type	RMSD (nm)	RMSF (nm)	RG (nm)	SASA (nm^2^)	Inter H-bond (nm)	Covariance (PCA)
PTPN11-WT-APO PTPN11-E76K-APO	0.24 ± 0.03	0.14 ± 0.08	2.55 ± 0.02	249.32 ± 3.81	-	41.25
	0.27 ± 0.05	0.14 ± 0.08	2.55 ± 0.01	245.58 ± 4.62	-	38.71
PTPN11-WT-aza PTPN11-E76K-aza	0.26 ± 0.05	0.13 ± 0.08	2.56 ± 0.03	246.18 ± 3.96	3.15 ± 2.19	31.23
	0.23 ± 0.02	0.12 ± 0.08	2.55 ± 0.01	248.29 ± 3.81	3.26 ± 1.20	31.50
PTPN11-WT-PD-901 PTPN11-E76K-PD-901	0.29 ± 0.05	0.12 ± 0.08	2.56 ± 0.01	241.39 ± 4.80	1.94 ± 1.06	33.02
	0.27 ± 0.05	0.16 ± 0.09	2.57 ± 0.02	246.21 ± 4.65	2.35 ± 1.04	31.68
PTPN11-WT-aza PD-901 PTPN11-E76K-aza-PD-901	0.26 ± 0.04	0.14 ± 0.09	2.58 ± 0.02	251.65 ± 3.88	4.21 ± 1.65	39.03
	0.25 ± 0.03	0.13 ± 0.09	2.59 ± 0.02	252.95 ± 4.22	3.06 ± 1.50 nm	38.42

WT, Wild Type; E76K - Mutant Type.

The PTPN11-WT-PD-901 and PTPN11-E76K-PD-901 complexes exhibited similar deviations from the beginning (0 ns) to ∼15 ns, then reached equilibrium within 50 ns and remained evenly distributed throughout the simulation (Figure 6C). The average RMSD values for PTPN11-WT-PD-901 and PTPN11-E76K-PD-901 were 0.29 ± 0.05 nm and 0.27 ± 0.05 nm, respectively, as shown in Table 2. The dual combination of PTPN11-WT-Aza-PD-901 and PTPN11-E76K-Aza-PD-901 complexes deviates from the beginning to ∼35 ns, then reaches equilibrium around ∼55 ns, and maintains this equilibrium until the end of the simulation (Figure 6D). The average RMSD values for PTPN11-WT-PD-901 and PTPN11-E76K-PD-901 were 0.26 ± 0.04 nm and 0.25 ± 0.03 nm, respectively, as shown in Table 2.

#### 3.2.2 RMSF

**FIGURE 7:**
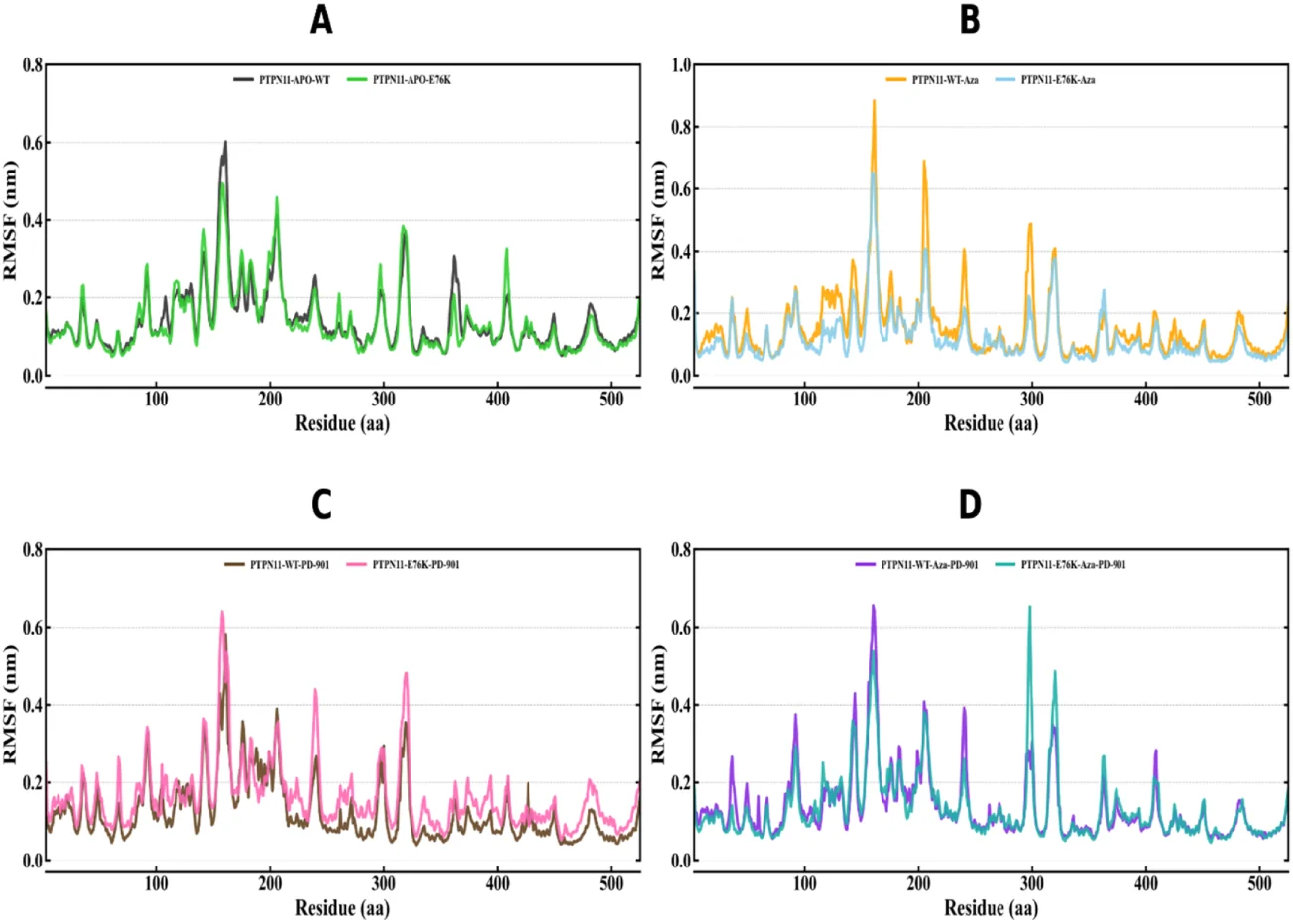
RMSF plot showing the residue-wise flexibility over the period of 300 ns. **(A)** PTPN11-APO-WT Vs. PTPN11-APO-E76K; **(B)** PTPN11-WT-Aza Vs. PTPN11-E76K-Aza; **(C)** PTPN11-WT-PD-901 Vs. PTPN11-E76K-PD-901; **(D)** PTPN11-WT-Aza-PD-901 Vs. PTPN11-E76K-Aza-PD-901.

Residue flexibility was analyzed by comparing the RMSF plots across all systems over the simulation time (Figures 7A–D). In the apo systems, PTPN11-APO-WT and PTPN11-APO-E76K, significant variation was observed across several regions, indicating greater flexibility in these regions (Figure 7A). The average RMSFs of these complexes were 0.14 ± 0.08 nm and 0.14 ± 0.08 nm, respectively (Table 2). The analysis showed that the PTPN11-WT-Aza and PTPN11-E76K-Aza complexes showed lower flexibility than the apo complexes (Figure 7B). The mean RMSF values for the WT and mutant complexes were 0.13 ± 0.08 nm and 0.12 ± 0.02 nm, respectively (Table 2). The PTPN11-WT-PD-901 and PTPN11-E76K-PD-901 complexes exhibited more stable conformations (Figure 7C). The average RMSF values for these two complexes were found to be 0.12 ± 0.08 nm for the wild-type complex and 0.16 ± 0.09 nm for the mutant complex (Table 2). PTPN11-WT-Aza-PD-901 and PTPN11-E76K-Aza-PD-901 combined complexes exhibited the most stable behavior throughout the simulation (Figure 7D). The RMSFs averaged 0.14 ± 0.09 nm for WT and 0.13 ± 0.09 nm for the MT complex (Table 2). Overall, a progressive reduction in residue-level fluctuations was observed from apo to monotherapy and further to combination therapy.

#### 3.2.3 Rg

**FIGURE 8:**
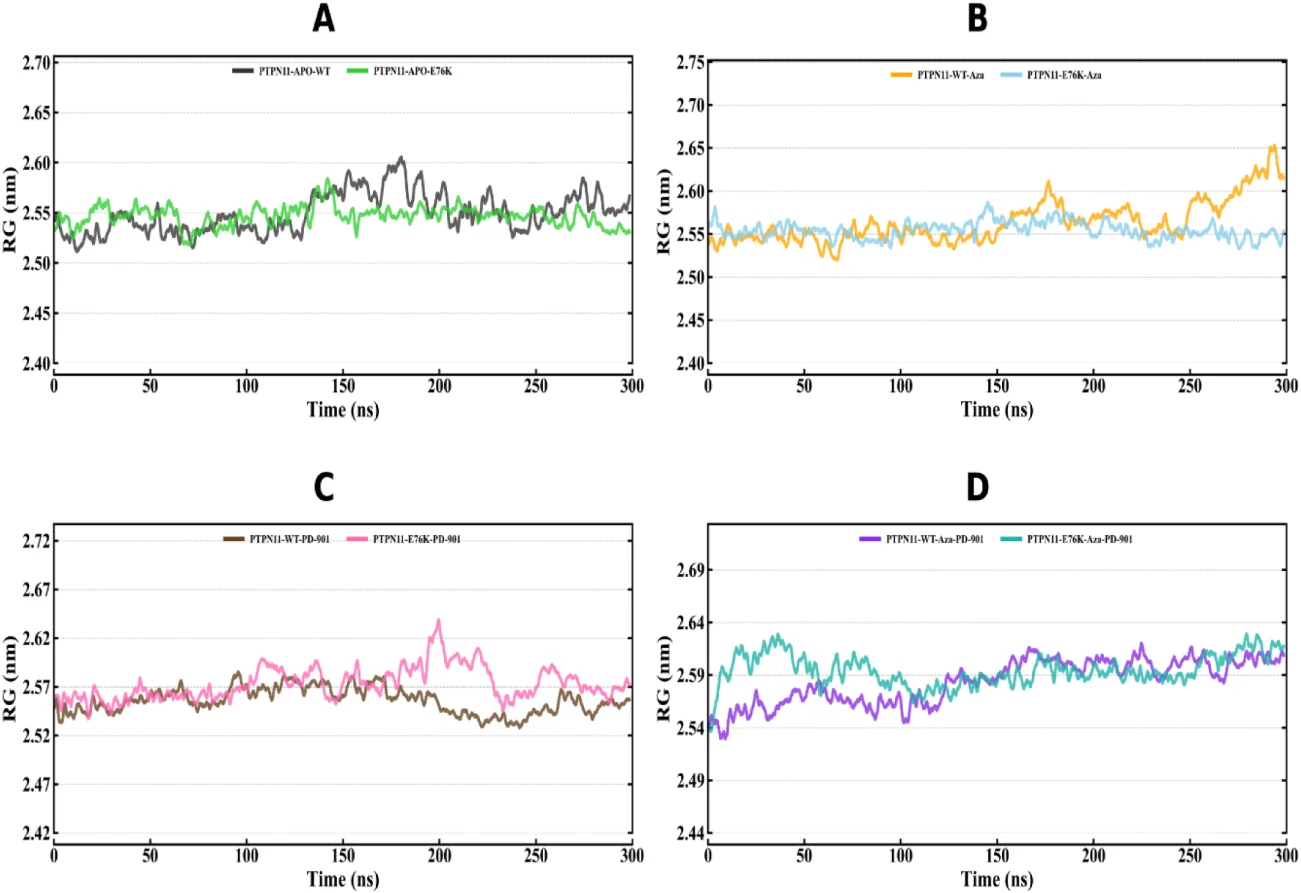
Rg distribution plot comparing the compactness over the period of 300 ns. **(A)** PTPN11-APO-WT Vs. PTPN11-APO-E76K; **(B)** PTPN11-WT-Aza Vs. PTPN11-E76K-Aza; **(C)** PTPN11-WT-PD-901 Vs. PTPN11-E76K-PD-901; **(D)** PTPN11-WT-Aza-PD-901 Vs. PTPN11-E76K-Aza-PD-901.

The degree of compactness of the protein complexes was quantified based on their radius of gyration (Rg) (Figures 8A–D). The apo complexes showed initial fluctuations, followed by stabilization, indicating modest compactness (Figure 8A). The calculated Rg of the apo complexes of PTPN11-APO-WT and PTPN11-APO-E76K were 2.55 ± 0.02 nm and 2.55 ± 0.01 nm, respectively (Table 2). The complexes with 5-azacitidine had stable Rg values after equilibrium (Figure 8B). The average Rg values of PTPN11-WT-Aza and PTPN11-E76K-Aza were 2.56 ± 0.03 nm and 2.55 ± 0.01 nm, respectively. The PD-901-bound systems exhibited uniformity in the Rg pattern, with no deviation (Figure 8C). The mean Rg values were 2.56 ± 0.01 nm and 2.57 ± 0.02 nm for WT and mutant, respectively (Table 2). In contrast, the PTPN11-WT-Aza-PD-901 and PTPN11-E76K-Aza-PD-901 combined complexes exhibited consistent Rg distribution patterns, with slight variation throughout the simulation (Figure 8D). The Rg values were 2.58 ± 0.02 nm and 2.59 ± 0.02 nm for WT and mutant, respectively (Table 2). This implies that all systems maintained compact structures; however, the combination systems showed increased compactness.

#### 3.2.4 SASA

**FIGURE 9:**
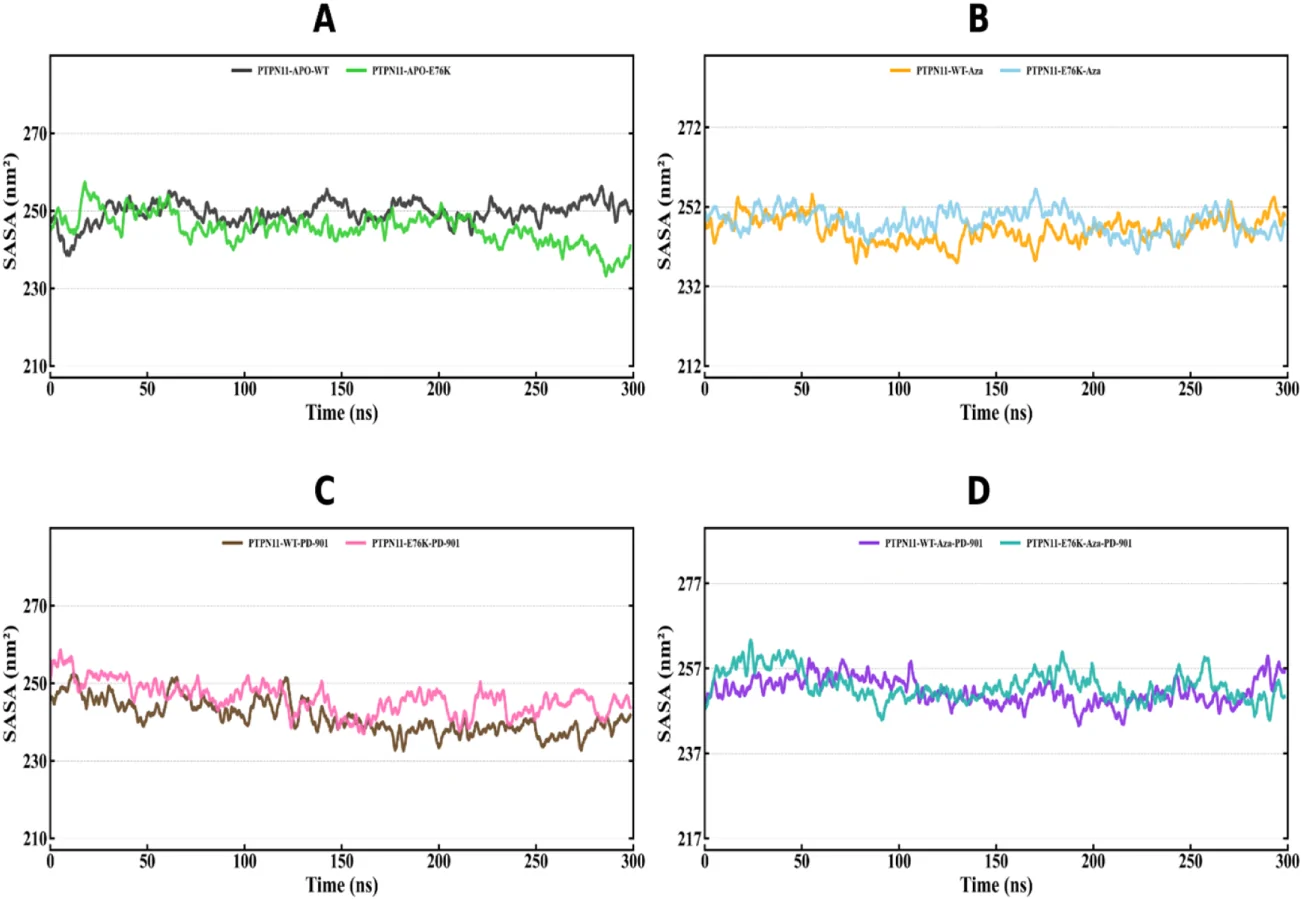
SASA plot comparing the solvent-accessible surface area over the period of 300 ns. **(A)** PTPN11-APO-WT Vs. PTPN11-APO-E76K; **(B)** PTPN11-WT-Aza Vs. PTPN11-E76K-Aza; **(C)** PTPN11-WT-PD-901 Vs. PTPN11-E76K-PD-901; **(D)** PTPN11-WT-Aza-PD-901 Vs. PTPN11-E76K-Aza-PD-901.

The effects on the protein’s surface accessibility were studied by SASA analysis (Figures 9A–D). It was observed that the SASA values of the apo structures changed considerably over the simulation time due to their dynamic interactions with the solvent molecules (Figure 9A). The average SASA values for PTPN11-APO-WT and PTPN11-APO-E76K were 249.32 ± 3.81 nm^2^ and 245.58 ± 4.62 nm^2^, respectively (Table 2). The 5-azacitidine-bound complexes showed lower SASA values, indicating reduced structural fluctuations and suggesting decreased conformational flexibility (Figure 9B). The SASA values for the WT and mutant complexes were 246.18 ± 3.96 nm^2^ and 248.29 ± 3.81 nm^2^, respectively (Table 2). On the other hand, SASA values in the PD-901-bound complexes were stable in nature (Figure 9C). The SASA values for the WT and mutant complexes were 241.39 ± 4.80 nm^2^ and 246.21 ± 4.65 nm^2^, respectively. The combined complexes showed the least SASA variation (Figure 9D). The mean SASA values were 251.65 ± 3.88 nm^2^ for the WT complex, whereas for the mutant complex, the mean SASA value was 252.95 ± 4.22 nm^2^ (Table 2). In general, SASA values increased progressively with ligand binding, especially in the combined complexes.

#### 3.2.5 Intermolecular hydrogen bond analysis

**FIGURE 10:**
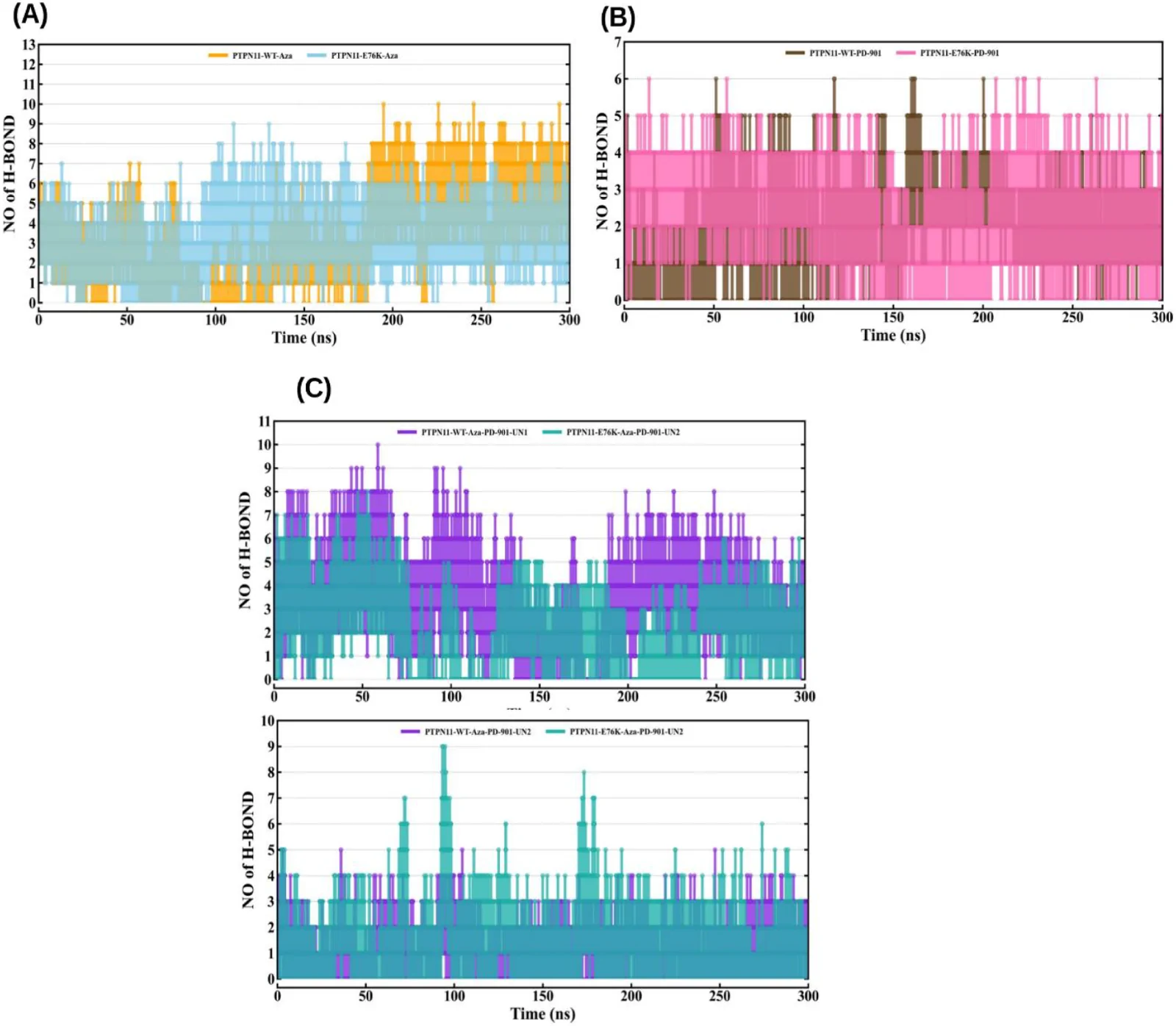
Intermolecular hydrogen bonds between protein-ligand during the simulation time (300 ns): **(A)** PTPN11-WT-Aza Vs. PTPN11-E76K-Aza; **(B)** PTPN11-WT-PD-901 Vs. PTPN11-E76K-PD-901; **(C)** PTPN11-WT-Aza-PD-901 Vs. PTPN11-E76K-Aza-PD-901.

Hydrogen-bond analyses were performed to identify intermolecular interactions for PTPN11-WT-Aza, PTPN11-E76K-Aza, PTPN11-WT-PD-901, PTPN11-E76K-PD-901, PTPN11-WT-Aza-PD-901 and PTPN11-E76K-Aza-PD-901 complex systems (Figures 10A–C). The apo forms of PTPN11-APO-WT and PTPN11-APO-E76K showed no intermolecular H-bonds in the absence of ligand. The PTPN11-WT-Aza and PTPN11-E76K-Aza complexes showed stable intermolecular H-bonds throughout the simulation (Figure 10A). The average number of intermolecular H-bonds was 3.15 ± 2.19 for WT and 3.26 ± 1.20 for the mutant complexes (Table 2). The PTPN11-WT-PD-901 and PTPN11-E76K-PD-901 complexes formed stable intermolecular H-bonds from the beginning to the end of the simulations (Figure 10B). The number of PTPN11-E76K-PD-901 intermolecular H-bonds was lower compared to PTPN11-WT-PD-901. The average values were 1.94 ± 1.06 for the WT and 2.35 ± 1.04 for the mutant (Table 2). On the other hand, the combination systems comprising PTPN11-WT-Aza-PD-901 and PTPN11-E76K-Aza-PD-901 complexes exhibited more intermolecular H-bonds than the monotherapy systems (Figure 10C). The average intermolecular hydrogen-bond value was 4.21 ± 1.65 for the WT and 3.06 ± 1.50 for the mutant (Table 2). However, intermolecular H-bonds did not change across all combinations and remained stable. The combination systems had relatively more intermolecular interactions.

#### 3.2.6 PCA

**FIGURE 11:**
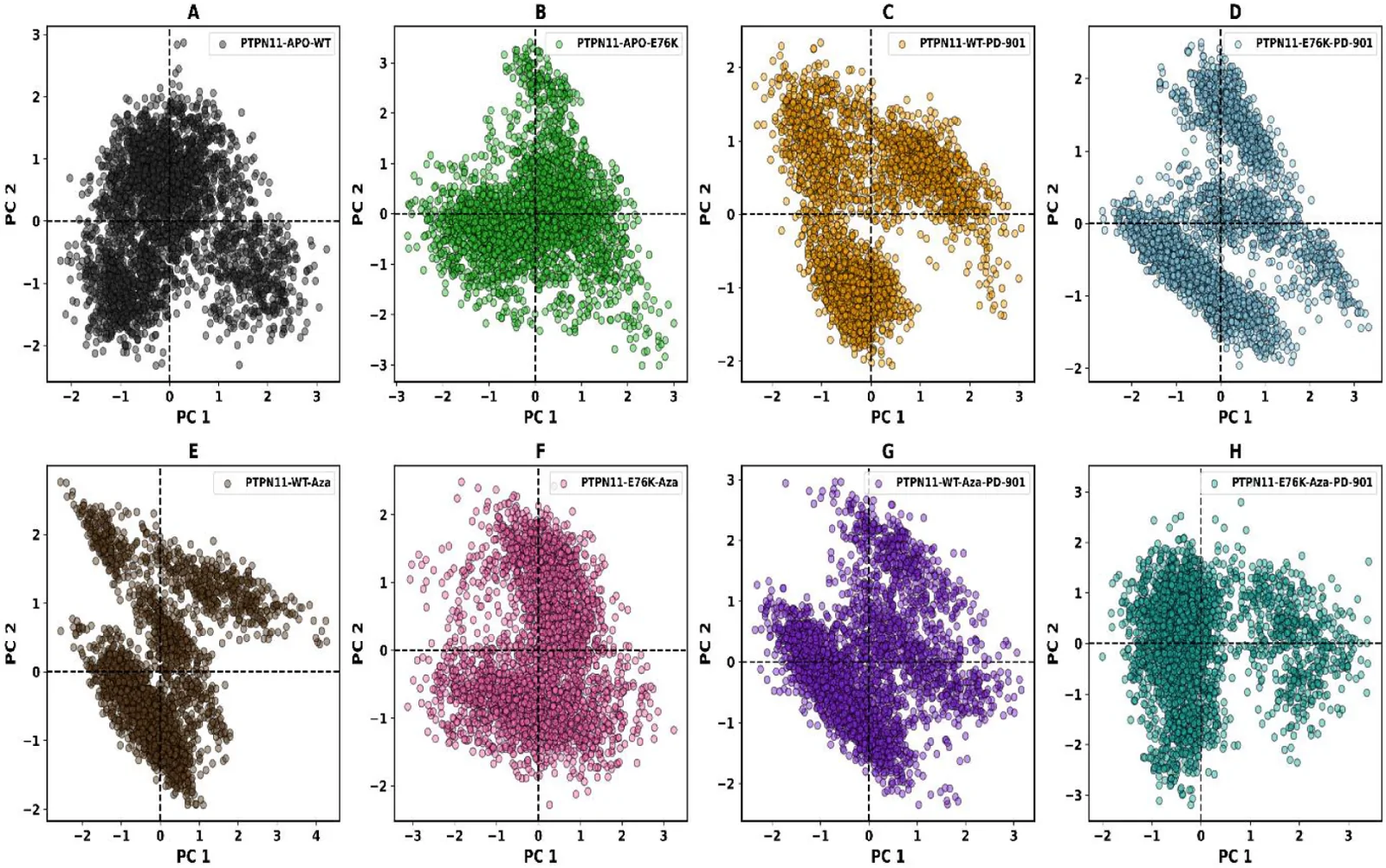
PCA plot illustrating the conformational dynamics of protein of **(A)** PTPN11-APO-WT, **(B)** PTPN11-APO-E76K, **(C)** PTPN11-WT-PD-901, **(D)** PTPN11-E76K-PD-901, **(E)** PTPN11-WT-Aza, **(F)** PTPN11-E76K-Aza,**(G)** PTPN11-WT-Aza-PD-901, and **(H)** PTPN11-E76K-Aza-PD-901.

The essential dynamics (PCA) analysis was performed to determine the conformational changes of the protein systems during the simulation (Figures 11A–H). The covariance values for the apo systems were 41.25 for PTPN11-WT-APO and 38.71 for PTPN11-E76K-APO (Table 2). The covariance of the bound complexes to 5-azacitidine was estimated to be 31.23 for PTPN11-WT-Aza and 31.50 for PTPN11-E76K-Aza (Table 2). On the other hand, when the complexed proteins were bound to PD-901, the covariance values were 33.02 and 31.68, respectively (Table 2). In the combination systems, the covariance values were 39.03 for PTPN11-WT-Aza-PD-901 and 38.42 for PTPN11-E76K-Aza-PD-901 (Table 2).

### 3.3 MM-PBSA and free energy calculation

To further assess the binding affinities of PTPN11-WT-Aza, PTPN11-E76K-Aza, PTPN11-WT-PD-901, PTPN11-E76K-PD-901, PTPN11-WT-Aza-PD-901 and PTPN11-E76K-Aza-PD-901 complexes, MM-PBSA calculations were carried out. The scoring function for MM-PBSA binding energies was obtained by taking the weighted sum of terms, including van der Waals interactions (vdW), Electrostatics (Elec), polar solvation, and Solvent-Accessible Surface Area (SASA). The binding free energy of the systems where 5-azacitidine binds at the active site was found to be −29.85 ± 16.41 kJ/mol in PTPN11-WT-Aza and −32.50 ± 13.07 kJ/mol in PTPN11-E76K-Aza (Table 3). However, the allosteric site-bound systems where PD-901 was the ligand gave binding affinities higher than those above at −86.14 ± 14.94 kJ/mol for PTPN11-WT-PD-901 and -72.67 ± 20.49 kJ/mol for PTPN11-E76K-PD-901 (Table 3). The binding free energies of PTPN11-WT-Aza-PD-901 and PTPN11-E76K-Aza-PD-901 combined complexes are higher than monotherapy, with values of −114.78 ± 30.34 and −138. 18 ± 23.91 kJ/mol, respectively (Table 3).

**TABLE 3:** MM-PBSA binding free energy values between apo & complex wild-type and mutant PTPN11 with 5-Azacitidine and PD-901.

Complex type	Binding energy (kJ/mol)	van der waals energy (kJ/mol)	Electrostatic energy (kJ/mol)	Polar solvation energy (kJ/mol)	SASA energy (kJ/mol)
PTPN11-WT-aza PTPN11-E76K-aza	−29.85 ± 16.41	−80.53 ± 6.68	−78.15 ± 41.96	138.52 ± 38.79	−9.69 ± 0.99
	−32.50 ± 13.07	−96.95 ± 15.31	−101.06 ± 13.03	177.87 ± 11.84	−12.36 ± 0.96
PTPN11-WT-PD-901 PTPN11-E76K-PD-901	−86.14 ± 14.94	−161.47 ± 15.14	−50.56 ± 18.11	143.14 ± 20.94	−17.26 ± 0.86
	−72.67 ± 20.49	−135.92 ± 16.08	−26.16 ± 21.46	104.65 ± 42.17	−15.23 ± 0.58
PTPN11-WT-aza-PD-901 PTPN11-E76K-aza-PD-901	−114.78 ± 30.34	−167.90 ± 40.27	−89.74 ± 61.96	163.63 ± 125.60	−20.76 ± 6.10
	−138. 18 ± 23.91	−224.65 ± 33.90	−47.09 ± 27.98	159.77 ± 63.54	−26.19 ± 4.22

WT, Wild Type; E76K - Mutant Type.

## 4 Discussion

Activation of the RAS/MAPK pathway plays a significant role in JMML due to gain-of-function mutations in PTPN11, especially the E76K mutation, which destabilizes the autoinhibitory structure of SHP2 leading to constitutive activation and proliferation of hematopoietic progenitor cells (Zhao et al., 2023; He et al., 2023; Zhang et al., 2025). With regard to this, the current research aimed to explore the possibility of enhancing inhibition efficiency through dual-site inhibition using a combination therapy approach rather than monotherapy. The use of docking, MD simulations, and MM/PBSA calculations in this study provides a logical rationale for analyzing these approaches.

The docking results provide clear distinctions in the binding efficacy of the drugs and their combinations. 5-Azacitidine (Aza), a drug primarily used, had relatively low binding affinities (−6.3 kcal/mol in the wild-type and −6.2 kcal/mol in the mutant). These weak interactions were expected for the compound since the main purpose of the drug is not to inhibit the SHP2 enzyme but to act on epigenetic modifications and gene expression (Wu et al., 2024; Sinha et al., 2023). On the other hand, PD0325901 (PD-901), an allosteric MEK inhibitor, had higher binding affinities (−8.6 kcal/mol in both wild-type and mutant forms). This finding is consistent with earlier studies indicating the importance of targeting the MAPK signaling pathway in PTPN11-induced leukemias (Pasupuleti et al., 2023; Chang et al., 2024; Liu et al., 2023). The commercially available MEK inhibitor, trametinib, had lower docking scores (−5.5 to −5.7 kcal/mol), indicating lower stability within the complex system.

One major insight from this analysis is the improvement in docking scores for the combination therapy systems. The 5-Aza and PD-901 combination scored −14.83 kcal/mol (WT) and −14.59 kcal/mol (E76K). Similarly, the 5-Aza and trametinib combination scored lower (−14 kcal/mol) but improved docking energies. The docking analysis demonstrated that 5-azacitidine and PD-901 could be accommodated simultaneously within the active and allosteric binding regions of PTPN11, respectively. However, direct quantitative comparison between single and dual-ligand docking scores should be interpreted cautiously since docking scores are best suited for ranking ligand poses within a specific binding site. Therefore, rather than suggesting combined binding effects, the docking results are mainly used to support the structural viability of simultaneous ligand binding. Instead, the ensuing molecular dynamics and MM-PBSA analyses, which revealed improved stability and favorable binding energetics, support the potential benefits of the dual-ligand systems. This observation supports numerous previous findings, indicating that using multiple targeted therapies could help overcome resistance in JMML treatments (Pasupuleti et al., 2023; Chang et al., 2024). Notably, the similar docking energies of WT and E76K mutants suggest that the combination therapy remains effective.

The MD simulations have further strengthened the docking predictions, showing that stable interactions have occurred in all protein-ligand complexes over 300 nanoseconds. The RMSD values for all the complexes ranged from ∼0.23 nm to ∼0.29 nm, indicating that the presence of the ligand does not make the protein complex unstable. The combination systems had lower RMSD values, ranging from ∼0.25 nm to ∼0.26 nm. This insight is significant, as stable binding over time is crucial for effective inhibition of signaling proteins such as SHP2 (Wu et al., 2021; Zhang et al., 2025). RMSF showed a distinct pattern in which apo systems exhibited higher fluctuations, whereas those bound to ligands were less flexible. Lower fluctuations were observed in the combination system. It suggests that ligand binding decreases conformational flexibility by constraining conformational movement, especially in loops that are crucial for the enzyme’s catalytic activity. This induced stability might hinder the conformational changes in PTPN11, especially in the E76K mutant (Zhang et al., 2025). Furthermore, this was supported by the results obtained from the RG and SASA studies. The overall structure of all the systems was found to be compact. Combination systems showed minimal variations in the Rg parameter, thus indicating good structural stability. Similarly, SASA values increased progressively with ligand binding, especially for the complexed systems.

The H-bonding analysis enhances an additional dimension of insight into the mechanism. The monotherapy systems showed a few intermolecular H-bonds (2–3), whereas the combination systems showed more intermolecular H-bonds (3–4), thereby forming stronger bonds. The formation of H-bonds is important because it helps form stronger bonds, which explains the improved docking results obtained for the combination systems. The PCA analysis revealed that ligand binding changes the conformational motion of PTPN11. The combination system showed stable movement with intermediate covariance values, indicating that large movements were constrained. This is especially pertinent to SHP2, whose activation requires conformational changes, which can be hindered by constraining large movements (Welsh et al., 2023; Kanumuri et al., 2022).

Finally, the MM-PBSA analysis quantitatively validated the efficacy of combination-based treatment. The binding energy for 5-Aza alone was rather low (−29.85 ± 16.41 kJ/mol for WT protein, −32.50 ± 13.07 for mutant), while PD-901 had much higher binding affinity (−86.14 kJ/mol for WT protein, −72.67 kJ/mol for mutant). In the combination system, even greater binding affinity to the protein was observed, −114.78 ± 30.34 kJ/mol for WT and −138. 18 ± 23.91 kJ/mol for the mutant, respectively. It is reasonable to assume that 5-Aza would promote allosteric binding of PD-901, and the observed binding properties might reflect improved stabilization of the protein-ligand complex. However, it is still necessary to conduct experiments to determine the connection between these computational results and functional inhibition. The results agree well with earlier studies indicating the potential effectiveness of combined treatments for JMML (Pasupuleti et al., 2023; Wu et al., 2024).

In summary, the current results show strong agreement between the MM-PBSA, molecular docking, and molecular dynamics simulation analyses. According to the findings, the combination of 5-azacitidine and PD-901 shows improved binding affinity, structural stability, and decreased conformational fluctuations. These computational studies support the hypothesis that simultaneous targeting of the active and allosteric regions of PTPN11 may influence protein conformational dynamics associated with SHP2 signalling. However, this study has several limitations. The analyses are based entirely on computational approaches and require experimental validation. Furthermore, the closed-state SHP2 structure was used to create the E76K mutant; independent replicate molecular dynamics simulations were not carried out; configurational entropy contributions were not taken into account in MM-PBSA calculations; and only the E76K mutation was studied. The results should therefore be interpreted in light of these limitations. However, this work offers a solid computational foundation for further experimental verification and the study of other clinically significant PTPN11 mutations.

## 5 Conclusion

This study provides a comprehensive computational investigation into the therapeutic targeting of PTPN11 in Juvenile Myelomonocytic Leukemia, with emphasis on the combined use of 5-Aza and PD-901. The results consistently demonstrate that although individual agents can interact with the protein and maintain structural stability, their inhibitory potential is comparatively limited when used independently. In contrast, the combination of 5-Aza and PD-901 shows a clear improvement in binding efficiency, interaction stability, and overall structural control in both wild-type and E76K-mutant systems. While molecular dynamics simulations and MM-PBSA analyses indicate that dual-ligand systems exhibit improved stability and favorable binding characteristics compared with the corresponding single-ligand complexes, molecular docking results confirm the structural viability of simultaneously targeting the active and allosteric sites. Hydrogen bond analysis also confirms more persistent and stable intermolecular interactions in the combination system, reflecting stronger ligand retention. Additionally, MM-PBSA free-energy calculations validate improved binding stability, particularly in the mutant protein, suggesting that the combination strategy is effective even in mutation-driven active conformations. Importantly, the dual-targeting approach appears to limit the conformational changes required for SHP2 activation, thereby providing a mechanistic advantage over monotherapy. These computational results suggest that combining 5-azacitidine and PD-901 may be a viable way of modifying the structural and dynamic characteristics of PTPN11. However, because these results are based on computational analyses, they do not show functional inhibition of SHP2 or therapeutic efficacy in JMML. As a result, the trends should be regarded as hypothesis-generating and further validated by experimental research. Hence, this work offers a computational framework that could assist future experimental studies and rational drug design initiatives targeting PTPN11-associated JMML.

## Data Availability

The original contributions presented in the study are included in the article/Supplementary Material, further inquiries can be directed to the corresponding authors.
